# STIM2 Mediates Excessive Store-Operated Calcium Entry in Patient-Specific iPSC-Derived Neurons Modeling a Juvenile Form of Huntington's Disease

**DOI:** 10.3389/fcell.2021.625231

**Published:** 2021-02-02

**Authors:** Vladimir A. Vigont, Dmitriy A. Grekhnev, Olga S. Lebedeva, Konstantin O. Gusev, Egor A. Volovikov, Anton Yu. Skopin, Alexandra N. Bogomazova, Lilia D. Shuvalova, Olga A. Zubkova, Ekaterina A. Khomyakova, Lyubov N. Glushankova, Sergey A. Klyushnikov, Sergey N. Illarioshkin, Maria A. Lagarkova, Elena V. Kaznacheyeva

**Affiliations:** ^1^Laboratory of Ionic Channels of Cell Membranes, Department of Molecular Physiology of the Cell, Institute of Cytology, Russian Academy of Sciences, St. Petersburg, Russia; ^2^Laboratory of Cell Biology, Department of Cell Biology, Federal Research and Clinical Center of Physical-Chemical Medicine, Federal Medical Biological Agency, Moscow, Russia; ^3^Center for Precision Genome Editing and Genetic Technologies for Biomedicine, Federal Research and Clinical Center of Physical-Chemical Medicine, Federal Medical Biological Agency, Moscow, Russia; ^4^Research Center of Neurology, Moscow, Russia

**Keywords:** calcium, store-operated calcium channels, Huntington's disease, induced pluripotent stem cells, neurodegeneration, EVP4593, STIM2

## Abstract

Huntington's disease (HD) is a severe autosomal-dominant neurodegenerative disorder caused by a mutation within a gene, encoding huntingtin protein. Here we have used the induced pluripotent stem cell technology to produce patient-specific terminally differentiated GABA-ergic medium spiny neurons modeling a juvenile form of HD (HD76). We have shown that calcium signaling is dramatically disturbed in HD76 neurons, specifically demonstrating higher levels of store-operated and voltage-gated calcium uptakes. However, comparing the HD76 neurons with the previously described low-repeat HD models, we have demonstrated that the severity of calcium signaling alterations does not depend on the length of the polyglutamine tract of the mutant huntingtin. Here we have also observed greater expression of huntingtin and an activator of store-operated calcium channels STIM2 in HD76 neurons. Since shRNA-mediated suppression of STIM2 decreased store-operated calcium uptake, we have speculated that high expression of STIM2 underlies the excessive entry through store-operated calcium channels in HD pathology. Moreover, a previously described potential anti-HD drug EVP4593 has been found to attenuate high levels of both huntingtin and STIM2 that may contribute to its neuroprotective effect. Our results are fully supportive in favor of the crucial role of calcium signaling deregulation in the HD pathogenesis and indicate that the cornerstone of excessive calcium uptake in HD-specific neurons is a calcium sensor and store-operated calcium channels activator STIM2, which should become a molecular target for medical treatment and novel neuroprotective drug development.

## Introduction

Since neurodegenerative disorders are one of the most acute and socially significant problems facing modern medicine, adequate models for these diseases are highly demanded. New perspectives in the modeling of hereditary neurodegenerative pathologies have arisen through patient-specific induced pluripotent stem cells (iPSCs) (Ishida et al., 2016; Mungenast et al., 2016; Nekrasov et al., 2016; Naphade et al., 2019).

Huntington's disease (HD) is a severe neurodegenerative pathology characterized by motor dysfunction, cognitive decline and the presence of mental disorders. At the molecular level, HD occurs due to an increase in the number of CAG repeats in the first exon of the gene encoding the huntingtin protein. The most vulnerable cells in HD are the striatal medium spiny neurons (Vonsattel and DiFiglia, 1998).

The involvement of disturbed intracellular calcium signaling in the pathogenesis of HD and other neurodegenerative diseases is widely discussed (Bezprozvanny, 2009; Wu et al., 2011; Egorova et al., 2015; Huang et al., 2016; Nekrasov et al., 2016; Czeredys et al., 2018; Hisatsune et al., 2018). Numerous potential drugs have demonstrated a pronounced specific effect on the pathological functioning of calcium signaling (Chen et al., 2011; Wu et al., 2011, 2016; Weber et al., 2019). The mutant huntingtin deregulates calcium signaling by many ways including interactions with mitochondria membranes (Panov et al., 2002; Choo et al., 2004) and calcium-binding proteins (Bao et al., 1996), impact on NMDA receptor trafficking (Fan et al., 2007), changes in the expression of genes responsible for calcium homeostasis (Luthi-Carter et al., 2002; Czeredys et al., 2013; Nekrasov et al., 2016) modulation of voltage-gated calcium channels activity (Silva et al., 2017; Chen et al., 2018). Moreover, mutant huntingtin was shown to interact with and potentiate the receptor for inositol-1,4,5-trisphosphate (InsP3R) thereby promoting calcium leakage from endoplasmic reticulum (ER) to cytosol (Tang et al., 2003, 2005).

The store-operated calcium (SOC) entry (SOCE) is one of the most ubiquitous pathways of calcium influx in mammalian cells, including neurons. SOCE physiologically occurs as a result of the InsP3R-mediated intracellular calcium store depletion and it is controlled by stromal interacting molecules (STIM1 and STIM2) – ER calcium sensors (Dziadek and Johnstone, 2007; Shalygin et al., 2015). The accumulated evidence indicates an important physiological role for neuronal SOCE both in normal and pathogenic conditions (Wegierski and Kuznicki, 2018). Several reports showed the importance of STIM2 for these processes (Ryazantseva et al., 2013; Sun et al., 2014; Wu et al., 2016; Yap et al., 2017; Czeredys et al., 2018). Alterations in SOCE were observed in various neurodegenerative pathologies (Ryazantseva et al., 2016, 2018; Secondo et al., 2018), including HD (Wu et al., 2011; Vigont et al., 2015, 2018; Nekrasov et al., 2016). Moreover, pharmacological inhibition of SOC channels have a neuroprotective effect in HD mice model YAC128 and improved motor functions in HD-afflicted flies (Wu et al., 2011). Also, RNAi knockdown or CRISPR/Cas9 knockout of different components of the SOC channels restore the density of spines in medium spiny neurons (MSNs) of YAC128 (Wu et al., 2018).

It has been established that the length of the polyglutamine (polyQ) tract in mutant huntingtin directly correlates with the severity of the disease and inversely correlates with the age of manifestation of the first symptoms for the vast majority of cases (Andrew et al., 1993; Illarioshkin et al., 1994). However, a few publications demonstrate some physiological cellular disturbances depending on the length of the polyQ tract (Ooi et al., 2019). In this paper, we investigated levels of store-operated and voltage-gated calcium uptakes in the newly developed patient-specific model of a juvenile form of HD (HD76) and compared this model with previously established low-repeat (40–47Q) HD models (Nekrasov et al., 2016). We also studied the role of STIM2 in SOCE alterations in HD76 neurons.

## Materials and Methods

### Ethical Approval and Patient Information

Primary skin fibroblasts were obtained from skin biopsies of the forearm of two healthy donors and one patient with HD (76Q repeats) as described in Chestkov et al. (2014). All donors signed informed consents (available upon request). Experiments were approved by ethical committees of Research Center of Neurology, Institute of Cytology RAS and Federal Research and Clinical Center of Physical-Chemical Medicine FMBA.

### Generation of iPSCs With Lentiviruses

We produced lentiviral particles based on LeGO vectors (Weber et al., 2010) containing Oct4, Sox2, Klf4, c-Myc (OSKM). We infected human skin fibroblasts at passage 3 with lentiviruses as described in Chestkov et al. (2014). 20–25 days after infection, iPSC-like colonies were mechanically passaged to separate wells of 48-well plates coated with Matrigel (Corning, USA) and cultivated as individual clones.

### iPSC Generation With Sendai Virus

Non-integrative reprogramming of human skin fibroblasts was made with CytoTune™-iPS 2.0 Sendai Reprogramming Kit (ThermoFisher Scientific, USA) according to manufacturer's instructions.

It should be noted that different approaches of iPSCs generation had no impact on calcium measurements performed in this study (in more details please see Results section The juvenile iPSCs-based HD model demonstrates pathologically enhanced SOCE and Supplementary Figure 1).

### iPSC Cultivation

We grew iPSCs in Petri dishes coated with Matrigel (Corning, USA). Up to the second passage, we cultured iPSCs in mTesR1 medium (Stemcell technologies, Canada) with 50 U/ml penicillin/streptomycin (Paneco, Russia). After the second passage, we cultured iPSCs in TesR-E8 medium (Stemcell technologies, Canada) with 50 U/ml penicillin/streptomycin (Paneco, Russia). We passaged iPSCs with 0.05% Trypsin (Hyclone, USA) and 5 μM Y27632 (Stemgent, USA) and cryopreserved iPSCs in FBS (Hyclone, USA) with addition of 10% DMSO (Paneco, Russia) and 5 μM Y27632 (Stemgent, USA).

In some experiments, we used iPSC-based models of HD (iPSHD22 with 47Q, iPSHD11 with 40Q, iPSHD34 with 42Q) and healthy iPSCs (UEF3B) described previously in Nekrasov et al. (2016) and Holmqvist et al. (2016).

The list of cell lines used in our study is presented in Supplementary Table 1.

### Embryoid Body Formation

We generated embryoid bodies (EB) from iPSCs with Aggrewell 400 system (Stemcell Technologies) according to manufacturer's protocol. After formation, EBs were transferred to Ultra-low-adhesion plates (Corning, USA) and cultured in DMEM/F12 (Paneco, Russia), 15% Knock-Out Serum Replacement (Invitrogen, USA), 5% FBS (Hyclone, USA), 0.1 mM β-mercaptoethanol (Sigma, USA), 1% NEAA (Hyclone, USA) and 50 U/ml penicillin-streptomycin (Paneco, Russia). The medium was changed every 2 days. Embryoid bodies were grown for 10 days, transferred to gelatin-coated Petri dishes and cultured for 15–20 days in the same medium.

### Karyotyping

We cultured iPSCs up to 80% confluency. Colcemid (0.2 μg/ml, Sigma) was added to cells 30 min before cell harvesting. The cells were washed two times with PBS (Paneco, Russia), detached with 0.05% Trypsin (Gibco, USA) and transferred to a conical tube with addition of 10% FBS (Hyclone, USA). Then 5 volumes of 0.075M KCl were added and the cell suspension was incubated for 10 min at 42°C. The cell suspension was then washed by centrifugation with fixatives (methanol: glacial acetic acid as 6:1 and 3:1). The resulting suspension was used for preparation of metaphase chromosomes. Karyotyping was performed using GTG-banding at a resolution of 400 bands with 20 metaphases analyzed. Metaphases were scored using Metafer semi-automated system and IKAROS software (MetaSystems GmbH, Altlussheim, Germany).

### Differentiation of iPSCs Into Neurons

We trypsinized iPSCs and plated at a density of 40,000 cells/cm^2^ in mTesR-1 medium (Stemcell technologies) in the presence of 5 μM ROCK inhibitor Y-27632 on a Matrigel substrate (Corning). Upon reaching a density of about 80–90%, cells were transferred to a medium for neuronal differentiation composed of: DMEM/F12 (Paneco, Russia), 1% N2 supplement (Paneco, Russia), 1 mM glutamine (Gibco, USA), 50 U/ml penicillin-streptomycin (Paneco, Russia), 200 nM LDN-193189, 10 μM SB431542 and 2 μM dorsomorphin (all from Miltenyi Biotec, USA). The medium was changed every 2 days; cells were cultivated for 14 days. The resulting neural precursors were split with Versene solution and incubated for 10 min in a CO2 incubator at 37°C. Cell suspension was centrifuged for 5 min at 300 g. Cells were plated at density of 250,000–400,000 cells per cm^2^ in Petri dishes or multi-well plates coated with Matrigel. The next day, the medium was changed to a medium for neural precursors composed of: Neurobasal (Invitrogen), 1% N2 supplement (Paneco, Russia), 1 mM glutamine (Gibco, USA), 50 U/ml penicillin-streptomycin (Paneco, Russia), 1 μM Purmorphamine, and 20 ng/ml FGF2 (Miltenyi Biotec, USA). The medium was changed every 2 days. Neural precursors were passaged once a week. At this stage, we had created a bank of cryopreserved neuronal progenitors. Neural precursors were cultured and frozen until the seventh passage. We reseeded neural precursors to a density of 300,000–400,000 cells per cm^2^. In order to obtain mature striatal-like GABAergic neurons, the cells were transferred to a medium for neuronal maturation consisting of: Neurobasal-A (Gibco, USA), 2% B27 supplement (Invitrogen, USA), 1 mM glutamine, 50 U/ml penicillin-streptomycin, 20 ng/ml BDNF, 20 ng/ml GDNF, and 2 μM forskolin (Miltenyi Biotec, USA). The medium was changed every other day. After 2 weeks, the concentration of BDNF and GDNF was reduced to 10 ng/ml, and after another week to 5 ng/ml. One month after the cells were transferred to the maturation medium, the obtained neurons were used in the experiments.

### PCR Analysis

The PCR primers used in the study are listed in Supplementary Table 2.

### Immunocytochemistry

Cells were grown on Petri dishes to a desired confluency. Then medium was aspirated and cells were washed with PBS (Paneco, Russia) and fixed in 4% PFA (Sigma, USA) for 20 min. PFA was aspirated and plates were washed with PBS and incubated in 0.1% Tween 20, (PBST) with addition of 0.3% Tryton-X100 for 10 min. Plates were washed with PBST three times. Cells were then incubated for 60 min in blocking solution consisting of PBST, 5% FBS (Hyclone, USA) and 5% Goat serum (Sigma, USA). Primary antibodies were then added in desired concentrations in blocking solution; cells were incubated overnight at 4°C. Primary antibodies were aspirated and cells were washed four times with PBST; secondary antibodies in PBST were added for 60 min. Cells were then washed three times with PBST. We added DAPI in a concentration of 100 ng/ml and cells were incubated for 2 min. Then DAPI was aspirated and cells were washed with PBS and visualized under a fluorescent microscope BX43 (Olympus, Japan). The list of antibodies is presented in Supplementary Table 3.

### Fluorescent Calcium Imaging

Cells were grown in 96-well cell culture plates (Corning, USA). Cells were loaded with 4 μM Fluo-4AM (Thermo Fisher Scientific) dye in the growth media and incubated at room temperature for 1 h. The cells were washed with HBSS (Hank's Balanced Salt Solution contained in mM: 130 NaCl, 2.5 KCl, 2 CaCl2, 1.2 MgCl2, 10 HEPES, and 10 glucose, pH was adjusted to 7.4 with NaOH) or HBSS without Ca2+ and containing 500 μM EGTA. The 96-well plates were then read on the FLUO star Omega Plate Reader (BMG Labtech) illuminated at 495 nm; a fluorescence emission was recorded at 510 nm. After a 3-s baseline read, we added to the microplate 1 μM of thapsigargin (Sigma, USA) prepared in HBSS buffer containing 2 or 4 mM CaCl_2_. Fluorescence results were calculated as the ratio of the fluorescence signal over the fluorescence signal before the addition of thapsigargin.

### Electrophysiological Studies

Ion currents were recorded using the whole-cell patch-clamp technique (Hamill and Sakmann, 1981). The measurements were made with an Axopatch 200B amplifier (Axon Instruments, USA). The microelectrode resistance was 5–10 MΩ; the series resistance was not compensated but continuously monitored throughout the experiment, with its values being in the range of 10–25 MΩ. The signal was enhanced and filtered with an internal 2-pole Bessel filter (section frequency 5,000 Hz) and digitized at 5,000 Hz using an AD converter plate (L-Card, Russia). To record currents through voltage-gated calcium channels, we maintained cells at −40 mV and applied a series of 500 ms voltage steps, from −80 to +50 mV with an increment of 10 mV. The pipette solution contained (in mM) 125 CsCl, 10 EGTA-Cs, 10 HEPES-Cs, 4.5 CaCl2, 1.5 MgCl2, 4 Mg-ATP, 0.4 Na-GTP pH was adjusted to 7.3 with CsOH. The extracellular solution contained (in mM) 140 NMDG-Asp, 10 BaCl2, 10 HEPES-Cs, 10 Glucose, pH was adjusted to 7.3 with CsOH. The recorded currents were normalized relative to cell capacitance (6–20 pF).

During the recording of integral currents through SOC channels, the membrane potential initially held at −40 mV. Then it was periodically (every 5 s) decreased to −100 mV for 30 ms, then gradually raised to 100 mV at a rate of 1 mV/ms, and then returned to −40 mV. Measurements were made at 0.5 mV intervals. The recorded currents were normalized relative to cell capacitance (6–20 pF). The traces recorded prior to current activation were used as templates for leak subtraction. Currents were evoked by the application of 1 μM thapsigargin to the external solution. The same solutions as for voltage-gated current recordings were used with addition of 0.01 mM nifedipine into the extracellular solution. All chemicals were obtained from Sigma-Aldrich (USA).

### Electrophoresis and Western Blotting

Cells were grown in 50-mm Petri dishes. After transfection, they were lysed in 10 mM Tris-HCl buffer (pH 7.5) with 150 mM NaCl, 1% Triton X-100, 1% NP40 (Nonidet P40, non-ionic detergent nonylphenoxypolyethoxylethanol), 2 mM EDTA, 0.2 mM phenylmethanesulfonylfluoride (PMSF; serine protease inhibitor), and protease inhibitor cocktail (Roche). Proteins were resolved by electrophoresis in 8% polyacrylamide gel and transferred to a PVDF membrane, pre-treated with methanol and transfer buffer (48 mM Tris, 39 mM glycine and 5% methanol). The membrane was incubated with 5% milk for 1 h at room temperature and treated with primary monoclonal anti-huntingtin antibody (1:5,000; catalog no. ab109115, Abcam, USA), or primary polyclonal anti-STIM2 antibody (1:1,000; catalog no. 4917, Cell Signaling Technology) and peroxidase-conjugated goat anti-mouse (1:30,000; catalog no. A0168, Sigma), or anti-rabbit (1:30,000; catalog no. A0545, Sigma, USA) IgG secondary antibody, respectively. Target proteins were visualized using the Super Signal West Femto Maximum Sensitivity Substrate (lot TH269871, Thermo Scientific, USA). All of the experiments were performed in at least three replications with different cell lysates. Monoclonal anti-α-tubulin antibody (1:1,000; catalog no. T6074, Sigma, USA) was used as the loading control. Relative protein content was estimated using standard software for comparing the intensity of bands in the scanned blots. The list of primary antibodies used in study is presented in Supplementary Table 3.

### Lentiviral Infection

Lentiviral particles were produced in HEK293T cells transfected with the plasmid for STIM2 suppression or control plasmid (described below), as well as MD2.G (AddGene no.12259, USA) and psPAX2 (AddGene no.12260, USA) packaging plasmid. The medium with lentiviral particles was harvested on the second and the third day post-transfection; viral particles were centrifuged (45,000 g) and resuspended in fresh medium. Viral particles were stored at −80°C. Neuronal cultures were infected using viral titers with a high level of transfection efficiency (no <90%).

To suppress STIM2, we used a plasmid encoding the shRNA against STIM2 (Sigma no. TRCN0000150821, sequence CCGGGCTCAATTTCAGACACTCATTCTCGAGAATGAGTGTCTGAAATTGAGCTTTTTTG in pLKO.1 vector). A non-target shRNA (cat. no SHC002, Sigma, USA) was used as a negative control.

### Statistics

Statistical analysis was performed using Origin 8.0 (Origin Lab). The amplitudes of currents were compared using one-way ANOVA (*p* < 0.05) with Bonferroni correction. The normality of the distribution and the equality of variances were evaluated using the Shapiro-Wilk and Leven tests, respectively. We used the Mann-Whitney-Wilcoxon test to compare the expression levels of the proteins.

## Results

### Establishment and Characterization of Human iPSCs

Human iPSC lines were established via reprogramming of primary fibroblasts of healthy donors and a patient with a juvenile form of HD by using either the lentiviral transduction method or Sendai viruses. We characterized iPSCs by their morphology, karyotype, expression of markers of pluripotent stem cells and ability to differentiate into derivatives of all three germ layers *in vitro*.

The iPSCs from diseased and healthy individuals were similar by these characteristics; all iPSCs had a normal karyotype, expressed TRA-1-60, SSEA4, Oct-4 and formed EBs upon spontaneous differentiation (Supplementary Figure 2).

### Characterization of Neuronal Populations

**Figure 1:**
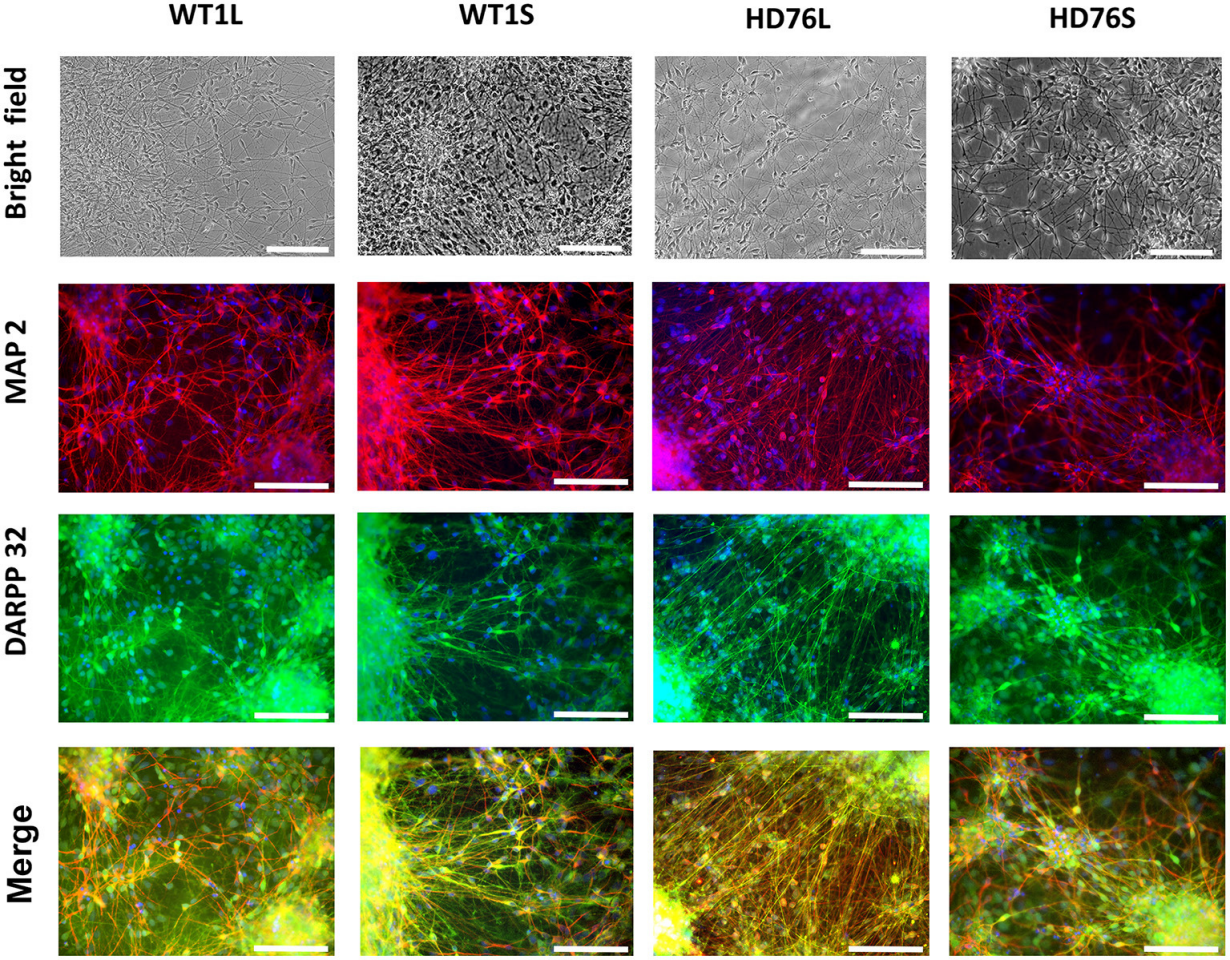
HD76 and WT iPSCs-derived neurons analysis. Representative images of neurons in the bright field (photo of the cells in culture) and immunostained for MAP2 (red), DARPP-32 (green) and merge of MAP2 and DARPP32 counterstained with DAPI (blue). HD76L and HD76S – juvenile HD-specific neuronal cell lines differentiated from iPSCs obtained by lentivirus and Sendai virus approaches, respectively; WT1L and WT1S – wild type neuronal cell lines differentiated from iPSCs obtained by Lentivirus and Sendai virus approaches, respectively. The cell lines are also represented in Supplementary Table 1. Scale bar 100 μm.

To create a fully physiologically adequate model, we produced terminally differentiated neuronal cells (striatal MSNs). To differentiate iPSCs into GABA-ergic MSNs, we used a protocol based on double inhibition of the SMAD cascade, followed by purmorphamine treatment directing cells into lateral ganglionic eminence progenitors (LGE) using purmorphamine, and further maturation of neurons with neurotrophic factors BDNF and GDNF. The LGE were differentiated into mature GABA MSNs during at least 20 days. Differentiated neurons had specific neuronal morphology; there were no visible differences between neurons derived from normal and mutant cells in terms of cell morphology, neurite length and viability. Differentiated neurons could be cultured for more than 3 months. Immunocytochemical analysis showed that up to 100% of the cells were specifically stained for neuronal marker MAP2 and up to 80% of MAP2-positive cells were specifically stained for DARPP-32, a known GABA MSNs specific marker (Figure 1).

### The Juvenile iPSCs-Based HD Model Demonstrates Pathologically Enhanced SOCE

Previously, we have demonstrated an augmented SOCE in 40–47 CAG repeats iPSC-based HD models. In all these described iPSCs-based models the amplitudes of SOCE were 2-fold higher compared to wild type GABA MSNs (Nekrasov et al., 2016). To address the question whether the amplitude of SOCE correlates with the length of the polyQ tract we performed the electrophysiological recordings of calcium currents in the newly established juvenile model of HD with 76Q (HD76).

To evoke store-operated calcium entry, we applied 1 μM thapsigargin; this blocks calcium pump SERCA and passively depletes intracellular calcium stores. For analysis, we subtracted the currents recorded prior to the activation of SOC channels. This protocol is a standard and ubiquitously used for SOC channels recordings in different cells (Kaznacheyeva et al., 2007; Wu et al., 2011; Pani et al., 2013; Czeredys et al., 2018; Lin et al., 2019) including patient-specific GABA MSNs (Nekrasov et al., 2016; Vigont et al., 2018).

**Figure 2:**
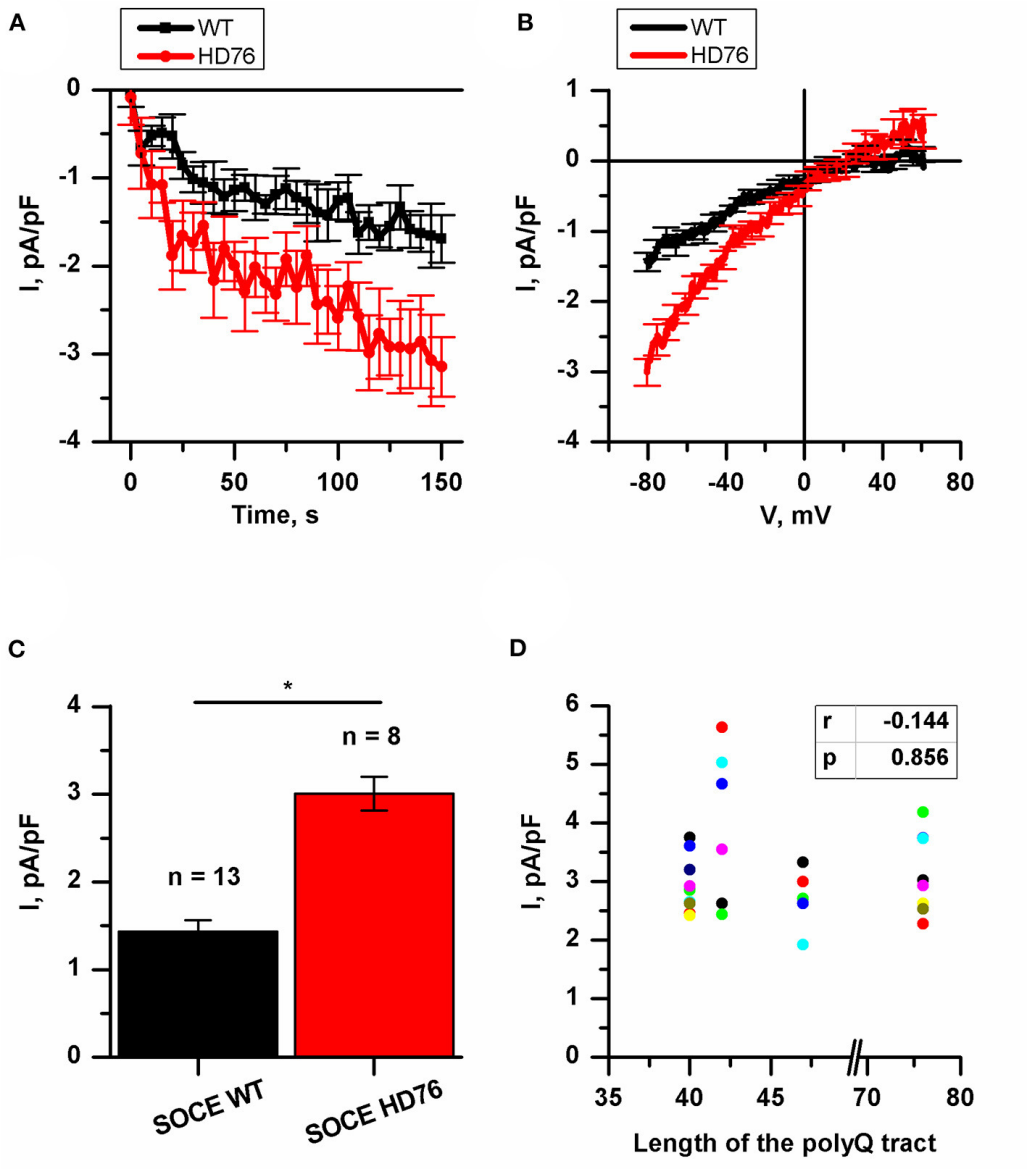
Disturbance of SOCE in HD76 neurons. **(A)** Normalized SOC currents evoked by application of thapsigargin (1 μM) and plotted as a function of time in HD76 (red circles), and WT (black squares) GABA MSNs. SOC currents were measured every 5 s at a test potential of −80 mV. Each trace shows mean ± SEM. **(B)** Average Current-Voltage relationships (I–V curves) of normalized currents evoked by passive depletion of calcium stores with thapsigargin (1 μM) in HD76 (red line), and WT (black line) GABA MSNs. The I–V curves were plotted after the full development of the SOC currents. The number of experiments is depicted at the panel **(C)**. **(C)** Average amplitude or the normalized SOC currents determined at a test potential of −80 mV for HD76 (red), and WT (black) GABA MSNs. The amplitudes are plotted as the mean ± SEM (*n* = number of single cell experiments). The asterisks indicate that differences in amplitudes are statistically significant (*p* < 0.05) **(D)** Correlation between the amplitude of SOC currents and the length of the polyglutamine tract of mutant huntingtin. The correlation coefficient (r) and the *p*-value are indicated above the plot. The cell lines are represented in Supplementary Table 1.

We showed that the peak amplitude of thapsigargin-induced calcium currents was 3.01 ± 0.19 pA/pF in HD76 neurons compared to 1.44 ± 0.13 pA/pF in wild-type neurons (WT GABA MSNs) obtained from healthy donors (Figures 2A–C). Taken together with previously published data (Nekrasov et al., 2016) our results demonstrate that SOCE is significantly higher in any HD-specific cell line including the juvenile HD model compared to any control cell line. At the same time, we did not see any correlation between SOCE amplitudes and the length of the polyQ tract of mutant huntingtin (Figure 2D).

It should be noted that in neuronal differentiation we used three different iPSCs from two patients as WT neurons and two different HD76 iPSCs from one patient which were reprogrammed using either the lentiviral transduction method or Sendai viruses. Comparing the electrophysiological features of iPSCs-derived neurons did not demonstrate any differences in SOCE neither for WT lines nor for HD76 neurons (Supplementary Figures 1A,B), so we combined the data from these lines and presented it as WT and HD76, respectively.

### EVP4593 Can Attenuate Pathologically Enhanced Level of Huntingtin in HD76 Neurons

It has been repeatedly demonstrated that mutant huntingtin has many toxic functions altering intracellular calcium signaling (Bao et al., 1996; Luthi-Carter et al., 2002; Panov et al., 2002; Tang et al., 2003, 2005; Choo et al., 2004; Fan et al., 2007; Czeredys et al., 2013; Silva et al., 2017; Chen et al., 2018), in particular, improper regulation of an activity of SOC channels (Wu et al., 2011; Vigont et al., 2015, 2018; Nekrasov et al., 2016).

**Figure 3:**
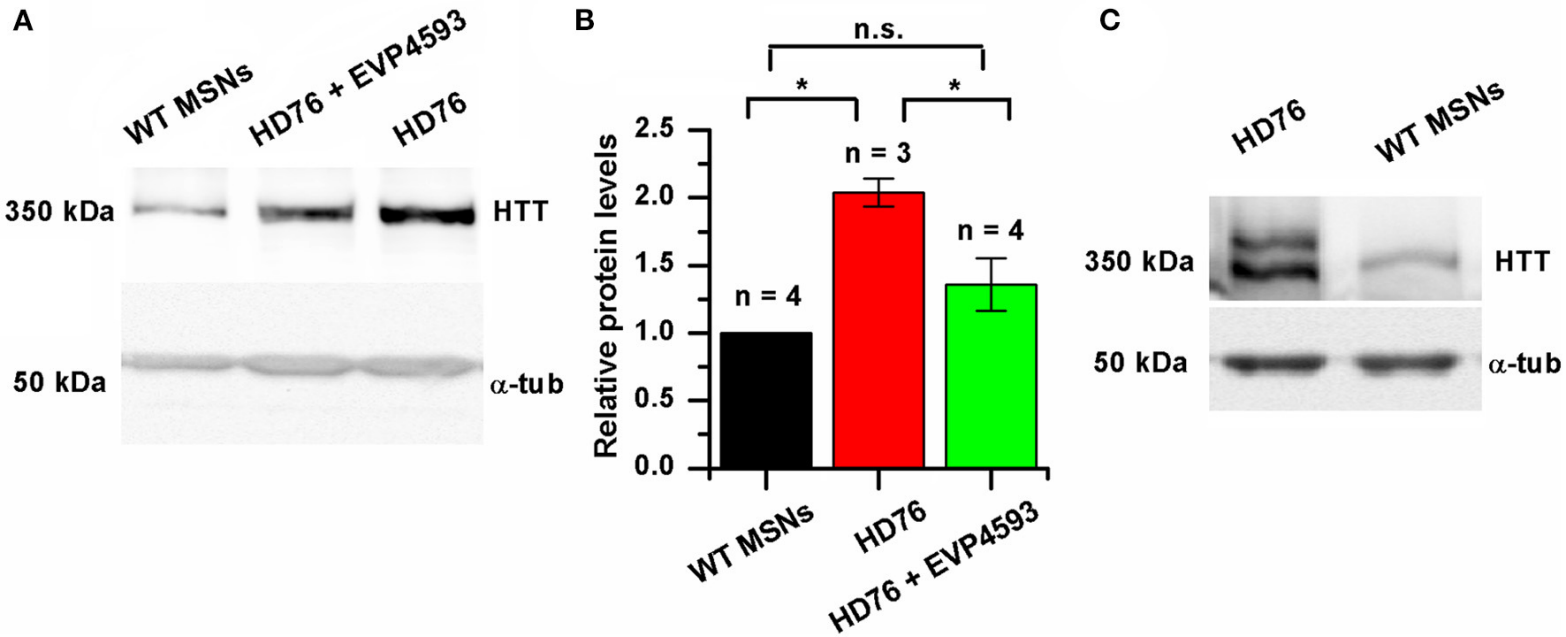
EVP4593 reduces the excessively increased level of mutant huntingtin. **(A)** Representative Western blot showing the expression level of total HTT in HD76, HD76 incubated with 300 nM EVP4593 for 24 h and WT GABA MSNs. Band intensities were quantified and values normalized to the α-tubulin housekeeping protein. **(B)** Relative HTT levels in WT (black), HD76 (red) and HD76 incubated with 300 nM EVP4593 for 24 h (green). Biological replicates are indicated above the bars. **(C)** Representative Western blot showing the expression level of normal and mutant HTT in HD76 and WT GABA MSNs. The asterisk indicates that differences in protein levels are statistically significant (*p* < 0.05).

We checked the level of expression of huntingtin and found it to be significantly greater in HD76 compared to WT GABA MSNs. Pre-incubation of the HD76 neurons with the previously described (Wu et al., 2011) potential anti-HD drug and SOC inhibitor EVP4593 in concentration of 300 nM for 24 hours before lysis decreased expression level of huntingtin, returning it to the level comparable with control values (Figures 3A,B). Thus, EVP4593 may be involved in the neuroprotection via regulation of expression of huntingtin.

HD76 neurons are specific for the heterozygous patient. In order to address if there are any preferences for huntingtin expression from normal or mutant alleles, we resolved the expression of normal (≪light≫) and mutant (≪heavy≫) huntingtin by long electrophoresis and Western blot analysis. Our data indicated that both normal and mutant huntingtin were overexpressed in HD76 neurons compared to WT GABA MSNs (Figure 3C). Moreover, we observed the same overexpression of huntingtin protein in neurons derived from HD iPSCs with shorter polyQ tract (Supplementary Figure 3A).

### STIM2 Mediates Excessive SOCE in HD76 Neurons

Currently, STIM proteins are established as well-known activators for SOC channels. In mammalians, this family is represented by two proteins: STIM1 and STIM2 (Dziadek and Johnstone, 2007). These proteins act as calcium sensors in the lumen of the ER and mediate the activity of SOC channels upon a loss of calcium binding. The STIM2 constant of calcium binding is lower than that of STIM1, so it can sense smaller changes in calcium ER content. Despite it being well-known that STIM2 is a relatively weak activator of SOCE, activation of the SOC channels by STIM2 is highly physiologically relevant. We have previously demonstrated that expression level of STIM2 was significantly higher in human neuroblastoma cells SK-N-SH modeling HD by overexpression of mutant huntingtin with 138 glutamine residues and huntingtin associated protein 1 (Czeredys et al., 2018).

**Figure 4:**
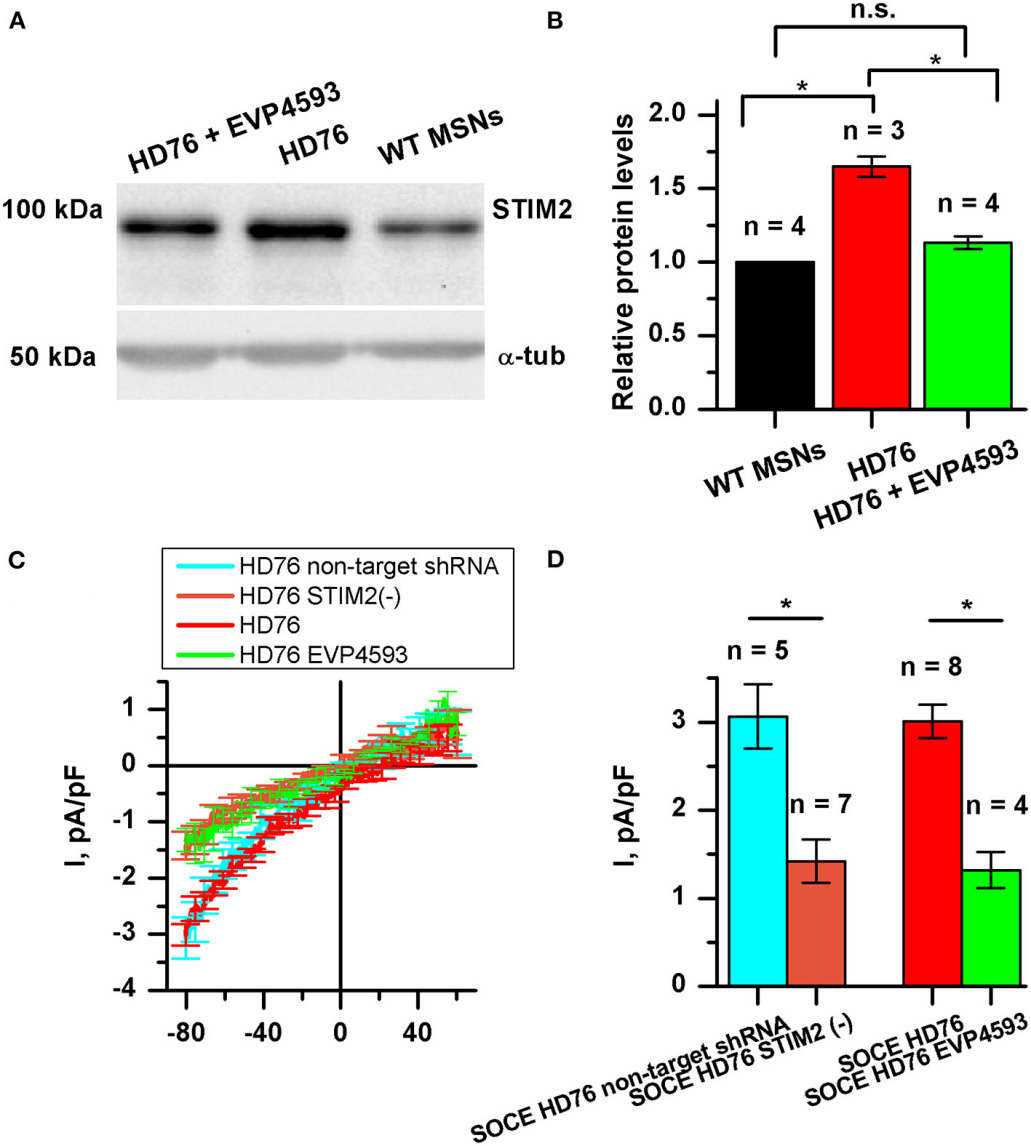
STIM2 as the cornerstone of significantly increased SOCE in HD76 GABA MSNs. **(A)** Representative Western blot showing the expression level of STIM2 in HD76, HD76 incubated with 300 nM EVP4593 for 24 h and WT GABA MSNs. Band intensities were quantified and values normalized to the α-tubulin housekeeping protein. **(B)** Relative STIM2 levels in HD76 (red), HD76 incubated with 300 nM EVP4593 (green) and WT GABA MSNs (black). Biological replicates are mentioned above the bars. **(C)** Average Current-Voltage relationships (I–V curves) of normalized currents evoked by passive depletion of calcium stores with thapsigargin (1 μM) in HD76 expressing non-target shRNA (HD76 non-target shRNA, cyan line), HD76 expressing shRNA against STIM2 (HD76 STIM2(–), orange line), intact HD76 (red line) and HD76 pre-incubated with 300 nM EVP4593 for 24 h (green line). The number of experiments is depicted at the panel **(D)**. **(D)** Average amplitude or the normalized SOC currents determined at a test potential of −80 mV for HD76 expressing non-target shRNA (cyan), HD76 expressing shRNA against STIM2 (orange), intact HD76 (red) and HD76 pre-incubated with 300 nM EVP4593 for 24 h (green). The amplitudes are plotted as the mean ± SEM (*n* = number of single cell experiments). The asterisk indicates that differences in amplitudes are statistically significant (*p* < 0.05).

We checked whether the STIM2 level is changed in HD GABA MSNs. The results of Western blot analysis demonstrated that in HD76, the level of STIM2 protein was 65% higher than in WT GABA MSNs (Figures 4A,B). Previously described low-repeat HD models also showed higher levels of STIM2 compared to WT neurons (Supplementary Figure 3B). Despite the fact that the gene encoding STIM2 has no NF-κB-dependent promoters or enhancers, EVP4593 surprisingly had an ability to attenuate the STIM2 level after 24 h incubation (Figures 4A,B).

Our findings allowed us to speculate that the excessive expression of STIM2 may underlie pathological SOCE in HD76. To test this hypothesis, we checked whether STIM2 suppression by EVP4593 (Figures 4A,B) affects calcium entry through SOC channels. The amplitude of thapsigargin-induced calcium currents in HD76 neurons after 24 h incubation with 300 nM EVP4593 was 1.32 ± 0.21 pA/pF (Figures 4C,D), demonstrating significantly lower level than in intact HD76 neurons (3.01 ± 0.19 pA/pF).

To verify the hypothesis that excessive SOCE in HD76 neurons depends on STIM2, we knocked down its expression in HD76 by lentiviral infection of shRNA against STIM2. These cells were named HD76 STIM2(–). The non-target shRNA was used as a negative control. After confirmation of STIM2 suppression by Western blot (Supplementary Figure 4), we registered store-operated calcium currents in HD76 STIM2(–). The amplitude of thapsigargin-induced calcium currents was 3.06 ± 0.37 pA/pF in HD76 non-target shRNA compared to 1.42 ± 0.24 pA/pF in HD76 STIM2(–) neurons (Figures 4C,D). Thus, we demonstrated that STIM2 suppression in HD76 neurons by using both shRNA-mediated knockdown and EVP4593 application leads to significant reduction of SOCE (Figures 4C,D). We also found that STIM2 suppression mediates SOC channels in WT GABA MSNs resulting in about 35% attenuation of SOCE in WT STIM2(–) neurons (Supplementary Figure 5).

We also confirmed the decrease in SOCE in HD76 STIM2(–) by fluorescent calcium imaging with Fluo-4AM. We incubated cells in a calcium-free bath containing 0.5 mM EGTA for 40 min to deplete the intracellular calcium stores and evoke activation of SOC channels. Then we returned calcium (final concentration was 4 mM) into the bath solution and added the thapsigargin to prevent refilling the stores. The measurements demonstrated that the steady-state level of the relative fluorescence of Fluo-4 after the SOC channels activation was 1.70 ± 0.07 a.u. in HD76 non-target shRNA compared to 1.47 ± 0.09 a.u. HD76 STIM2(–) (Supplementary Figure 6); this confirmed the data obtained by electrophysiological recordings.

To further strengthen the conclusion that STIM2 drives the enhanced SOCE in HD76 neurons we performed experiments with G418, which is known to be a selective antagonist of STIM2. Electrophysiological recordings showed that application of 50 mkM of G418 decreased SOCE amplitude in HD76 neurons from 3.27± 0.53 to 1.23 ± 0.36 pA/pF (Supplementary Figure 7).

Altogether our data indicate that STIM2 drives pathological hyperactivity of SOC channels in HD76 neurons.

### HD76 Neurons Demonstrate Upregulated Calcium Entry Through Voltage-Gated Channels

**Figure 5:**
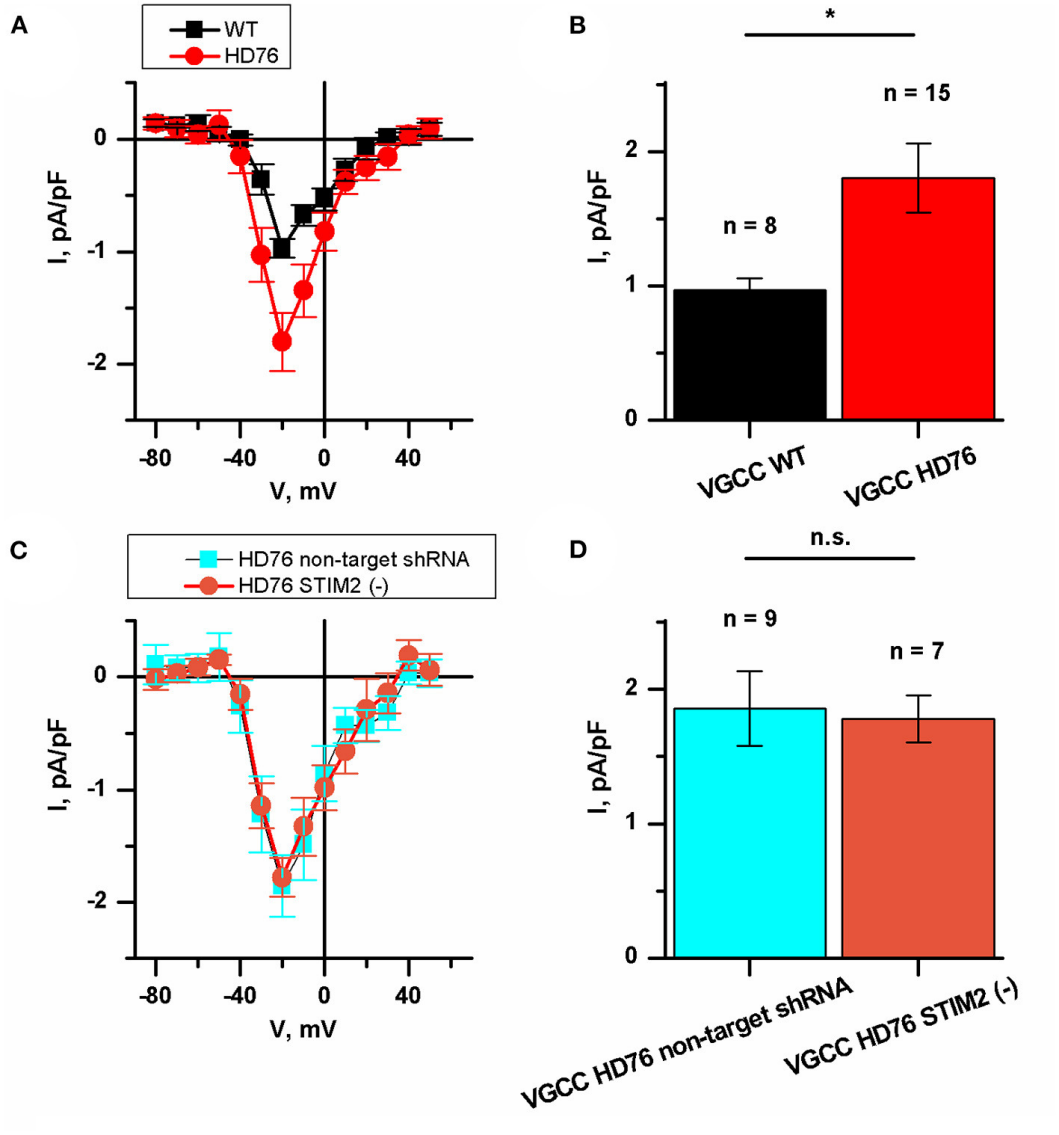
Disturbance of VGCC in HD76 neurons. **(A)** Average I–V curves of normalized voltage-gated calcium currents for HD76 (red circles) and WT (black squares) GABA MSNs. The number of experiments is depicted at the panel **(B)**. **(B)** Average amplitude of VGCC currents at the potential of −20 mV for HD76 (red), and WT (black) GABA MSNs. The amplitudes are plotted as the mean ± SEM (*n* = number of single cell experiments). The asterisk indicates that differences in amplitudes are statistically significant (*p* < 0.05). **(C)** Average I–V curves of normalized voltage-gated calcium currents in HD76 expressing non-target shRNA (HD76 non-target shRNA, cyan squares) and HD76 expressing shRNA against STIM2 (HD76 STIM2(–), orange circles). The number of experiments is depicted at the panel **(D)**. **(D)** Average amplitude of VGCC currents at the potential of −20 mV for HD76 expressing non-target shRNA (HD76 non-target shRNA, cyan) and HD76 expressing shRNA against STIM2 (HD76 STIM2(–), orange). The amplitudes are plotted as the mean ± SEM (*n* = number of single cell experiments). n.s. indicates the absence of statistically significant differences (*p* > 0.05).

We also measured the currents through the voltage-gated calcium channels (VGCC) in HD76 neurons. The peak amplitudes of VGCC current in HD76 and WT GABA MSNs were 1.80 ± 0.26 and 0.97 ± 0.08 pA/pF, respectively (Figures 5A,B); This demonstrates a pathological increase of calcium entry through VGCC by ~85%. Moreover, we detected analogical increase in voltage-gated calcium uptake in low-repeat HD model (Supplementary Figure 8). These currents were partially sensitive to L-type channels blocker nifedipine (Supplementary Figure 9). As well as for SOCE, we also confirmed that amplitudes of currents through VGCC do not significantly vary in different WT and HD76 lines (Supplementary Figures 1C,D).

Then we showed that STIM2 suppression had no effect on calcium entry through VGCC. The peak amplitude of VGCC current in HD76 non-target shRNA was 1.86 ± 0.28 pA/pF compared to 1.78 ± 0.17 pA/pF in HD76 STIM2(–) neurons (Figures 5C,D). Thus, we concluded that the upregulation of VGCC in HD76 neurons is not associated with high STIM2 levels.

## Discussion

Previously we have characterized iPSC-based low-repeat HD models and postulated that SOCE is dramatically upregulated in HD-specific GABA MSNs (Nekrasov et al., 2016). Here we measured SOC currents in HD76 neurons, modeling a juvenile form of HD. Since it is well-known that the severity of HD pathology highly correlates with the length of the polyQ tract of mutant huntingtin, we expected to find differences in calcium disturbances between low-repeat and juvenile models. However, our data indicate that SOCE in HD-specific GABA MSNs does not depend on the length of the polyQ tract. One possible hypothesis explaining this is that the cells have a limit to channel-forming and/or activator proteins. Indeed, in a previously described human neuroblastoma SK-N-SH HD model, we expected that overexpression of Hap1 protein (huntingtin associated protein 1) resulted in further SOCE increase, but we have not confirmed this effect since all SOC channels had already been activated upon mutant huntingtin expression (Czeredys et al., 2018). However, it has recently been found that isogenic HD-specific neural cells exhibit mitochondrial deficits and high levels of reactive oxygen species, correlating with the length of the polyQ tract (Ooi et al., 2019). Thus, we conclude that alterations in calcium signaling observed in cell models of HD are independent of the length of the polyQ tract and can be a common part of the pathogenesis of juvenile and late-onset forms of HD. However, mitochondrial impairments and high levels of reactive oxygen species can be caused by processes located downstream of calcium uptake and demonstrate a strong correlation with the length of the polyQ tract.

A number of studies of iPSC-based HD models were focused on alterations in gene expression. Mostly, the RNAseq technique is used to determine the upregulated or downregulated genes in HD-specific cells. Such investigations are highly relevant since it provides novel candidates for medical treatment. A number of scientific groups observed differential gene expression in iPSC-based HD models (HD iPSC Consortium, 2017; Switońska et al., 2018; Ooi et al., 2019). We previously reported a gene ontology analysis in low-repeat HD models indicating that genes maintaining calcium signaling are upregulated in HD GABA MSNs compared to WT GABA MSNs (Nekrasov et al., 2016).

In this paper, we showed that huntingtin expression in HD76 neurons is significantly higher at the protein level (Figures 3A,B). One of the translation-enhancing mechanisms is triggered by association of the expanded CAG repeat to RNA binding protein MID1, which recruits 40S ribosome kinase S6K to mCAG-RNA, and stimulates enhanced translation of mutant RNA (Krauss et al., 2013).

Since HD is a monogenic disease, the only way to totally treat it is to prevent the expression of the mutant huntingtin. One of the rapidly developing therapeutic approaches is using antisense oligonucleotide technology to lower huntingtin levels in HD (Aslesh and Yokota, 2020; Marxreiter et al., 2020). However, the searching for the small molecules able to supplement and/or enhance action of antisense oligonucleotides remains highly actual. Here we found that the potential anti-HD drug EVP4593 can attenuate high expression of the mutant huntingtin, thus, suppressing its toxic functions (Figures 3A,B). This compound was initially described as an inhibitor of activation of the NF-κB signal pathway (Tobe et al., 2003). Then we showed that EVP4593 can attenuate SOCE (Wu et al., 2011; Vigont et al., 2015) and now it is well accepted as a SOC channels inhibitor (Nekrasov et al., 2016; Vigont et al., 2018; Chernyuk et al., 2019). This compound has been previously tested on various HD models, including patient-specific GABA MSNs, demonstrating both SOCE inhibitory activity (Wu et al., 2011; Vigont et al., 2015, 2018; Nekrasov et al., 2016) and neuroprotective effect (Wu et al., 2011, 2016; Nekrasov et al., 2016). Despite it has been reported that EVP4593 specifically inhibit mitochondrial complex I (Krishnathas et al., 2017), the molecular mechanisms explaining its inhibition of SOC channels and neuroprotection remain unclear.

Our data allowed us to suppose that the neuroprotective effect of EVP4593 observed in HD-specific cells can be explained by the indirect impact of EVP4593 on the mutant huntingtin expression level. The effect of EVP4593 on huntingtin level was highly estimated since the gene encoding huntingtin has an NF-κB-dependent promoter/enhancer (Bečanović et al., 2015).

Earlier, it has been reported the tendency to shorter life-time of mutant huntingtin compared to normal huntingtin (Tsvetkov et al., 2013). This can explain the observed strong reduction in huntingtin level in HD76 neurons even after 24 h treatment with EVP4593 (Figures 3A,B). It has also been shown that in HD neurons derived from hESCs even 10% reduction of the mutant huntingtin level was sufficient to prevent toxicity, whereas up to 90% reduction of the wild-type huntingtin was tolerated and safe to those cells (Lu and Palacino, 2013). Hence, EVP4593-mediated attenuation of both normal and mutant huntingtin could have a potential clinical application in HD treatment.

We have demonstrated that high SOCE is a well-repeated parameter in HD-specific GABA MSNs and could be considered as a pathology marker. We also suggest that STIM2 is a promising target for drug design. We showed that STIM2 is upregulated in HD76 neurons and the pre-incubation of HD76 neurons with EVP4593 resulted in lowering the STIM2 level (Figures 4A,B). This result correlates well with the published data, demonstrating the attenuation of both lithium-induced and normal STIM2 expression by application of another NF-κB antagonist wogonin (Sukkar et al., 2018). Then we hypothesized that STIM2 drives the high activity of SOC channels in HD. Our data indicated that suppression of STIM2 in HD76 neurons using both shRNA-mediated knockdown and treatment with EVP4593 results in a significant reduction of pathological SOCE (Figures 4C,D). Furthermore, application of STIM2 antagonist G418 (Parvez et al., 2008) also reduced SOCE level in HD76 neurons (Supplementary Figure 7). The major role for STIM2 in HD is also supported by the study demonstrating STIM2-mediated dendritic spine dysregulation in neuron cultures of YAC128 HD mice (Wu et al., 2016).

Involvement of VGCC in HD pathogenesis was demonstrated in only few recent papers. They showed an important role for N-type VGCC in the increased release of synaptic vesicles at presynaptic terminals of HD cortical neurons in heterozygous zQ175 mice (Chen et al., 2018) and disease stage-dependent alterations in calcium influx in BACHD mice (Silva et al., 2017). A significant increase in L-type calcium currents in cortical neurons from BACHD mice has been also reported (Miranda et al., 2019). Here we have also shown greater calcium entry through VGCC in HD neurons. The recorded currents were partially sensitive to L-type VGCC blocker nifedipine (Supplementary Figure 7). Surprisingly we found that currents through VGCC were upregulated in HD76 under STIM2 suppression (Figures 5C,D). This result was unexpected since a reduction of STIM2 expression level may shift the balance to predominant activation of STIM1, which is known to be an inhibitor of the L-type VGCC (Park et al., 2010). Further investigations in the framework of the separate study are required to shed light on the molecular mechanisms of VGCC alterations in HD pathogenesis, their possible relation to SOCE pathway, and impacts of VGCC of different types.

In summary, the polyQ expansion within huntingtin affects neuronal calcium signaling in HD76 neurons, demonstrating high levels of calcium influx through both SOC channels and VGCC. Modulation of calcium channels and regulation of expression levels of proteins responsible for calcium uptake are perspective directions for anti-neurodegenerative drug development. We postulate that calcium sensor STIM2 drives a hyperactivity of the SOC channels in HD76 neurons, thus, establishing STIM2 as a promising molecular target for medical treatment. Further investigation will contribute to deeper insights into the molecular mechanisms of neurodegeneration. This hopefully will shed light on the key points of precision regulation of pathological intracellular signaling and contribute to patient-oriented personalized strategies in medical treatment.

## Data Availability Statement

The original contributions presented in the study are included in the article/Supplementary Material, further inquiries can be directed to the corresponding authors.

## Ethics Statement

The studies involving human participants were reviewed and approved by the ethical committee of the Research Center of Neurology, Moscow, Russian Federation the ethical committee of the Institute of Cytology RAS, Saint-Petersburg, Russian Federation the ethical committee of the Federal Research, and Clinical Center of Physical-Chemical Medicine FMBA, Moscow, Russian Federation. The patients/participants provided their written informed consent to participate in this study.

## Author Contributions

VV: conceptualization, methodology, validation, formal analysis, investigation, writing—original draft, writing—review and editing, visualization, project administration, and funding acquisition. DG: validation, formal analysis, investigation, writing—original draft, writing—review and editing, and visualization. OL: validation, formal analysis, investigation, writing—original draft, and writing—review and editing, visualization. KG: investigation. EV: investigation. AS: investigation. AB: formal analysis, investigation, and writing—review and editing. LS: investigation. OZ: investigation. EAK: investigation. LG: investigation. SK: investigation and resources. SI: conceptualization and resources. ML: conceptualization, methodology, formal analysis, writing—review and editing, visualization, supervision, project administration, and funding acquisition. EVK: conceptualization, methodology, formal analysis, writing—review and editing, visualization, supervision, project administration, and funding acquisition. All authors have read and agreed to the published version of the manuscript.

## Conflict of Interest

The authors declare that the research was conducted in the absence of any commercial or financial relationships that could be construed as a potential conflict of interest.

## Abbreviations

ER: endoplasmic reticulum
EVP4593: 4-N-[2-(4-Phenoxyphenyl)ethyl]quinazoline-4,6-diamine
HD: Huntington's disease
HD76: patient-specific neuronal model of juvenile form of Huntington's disease
HD76 STIM2(–): HD76 neurons with shRNA-mediated suppression of STIM2
iPSCs: induced pluripotent stem cells
MSNs: medium spiny neurons
polyQ: polyglutamine
SOC(E): store-operated calcium (entry)
VGCC: voltage-gated calcium channels
WT: wild type.
