# Supplementary material for: STIM2 Mediates Excessive Store-Operated Calcium Entry in Patient-Specific iPSC-Derived Neurons Modeling a Juvenile Form of Huntington's Disease
Source: Front Cell Dev Biol. 2021 Feb 2;9:625231. doi: 10.3389/fcell.2021.625231 (PMC7884642; doi:10.3389/fcell.2021.625231)
Supplement: Supplementary file 1 [file Data_Sheet_1.PDF]

Additional file 1.pdf

Supplementary material for *Frontiers in cell and developmental biology*

STIM2 mediates excessive store-operated calcium entry in patient-specific iPSC-derived neurons modeling a juvenile form of Huntington's disease

Vladimir A. Vigont[1,#], Dmitriy A. Grekhnev[1,#], Olga S. Lebedeva[2,3,#], Konstantin O. Gusev[1], Egor A. Volovikov[2], Anton Yu. Skopin[1], Alexandra N. Bogomazova[2,3], Lilia D. Shuvalova[2], Olga A. Zubkova [2], Ekaterina A. Khomyakova[2], Lyubov N. Glushankova[1], Sergey A. Klyushnikov[4], Sergey N. Illarioshkin[4], Maria A. Lagarkova[2,3,*], Elena V. Kaznacheyeva[1,*]

[1] Institute of Cytology, Russian Academy of Sciences, 4 Tikhoretsky Ave., St. Petersburg, 194064 Russia; cellbio@incras.ru

[2] Federal Research and Clinical Center of Physical-Chemical Medicine, Federal Medical-Biological Agency, 1a Malaya Pirogovskaya St., Moscow, 119435 Russia

[3] Center for Precision Genome Editing and Genetic Technologies for Biomedicine, Federal Research and Clinical Center of Physical-Chemical Medicine of Federal Medical Biological Agency, 1a Malaya Pirogovskaya St., Moscow, 119435 Russia

[4] Research Center of Neurology, 80 Volokolamskoe Hwy., Moscow, 125367 Russia

[#] These authors contributed equally to this work

[*] Correspondence:

evkazn@incras.ru (Elena Kaznacheyeva), Institute of Cytology, Russian Academy of Sciences, 4 Tikhoretsky Ave., St. Petersburg, 194064 Russia;

lagar@rcpcm.org (Maria Lagarkova), Federal Research and Clinical Center of Physical-Chemical Medicine, Federal Medical-Biological Agency, 1a Malaya Pirogovskaya St., Moscow, 119435 Russia

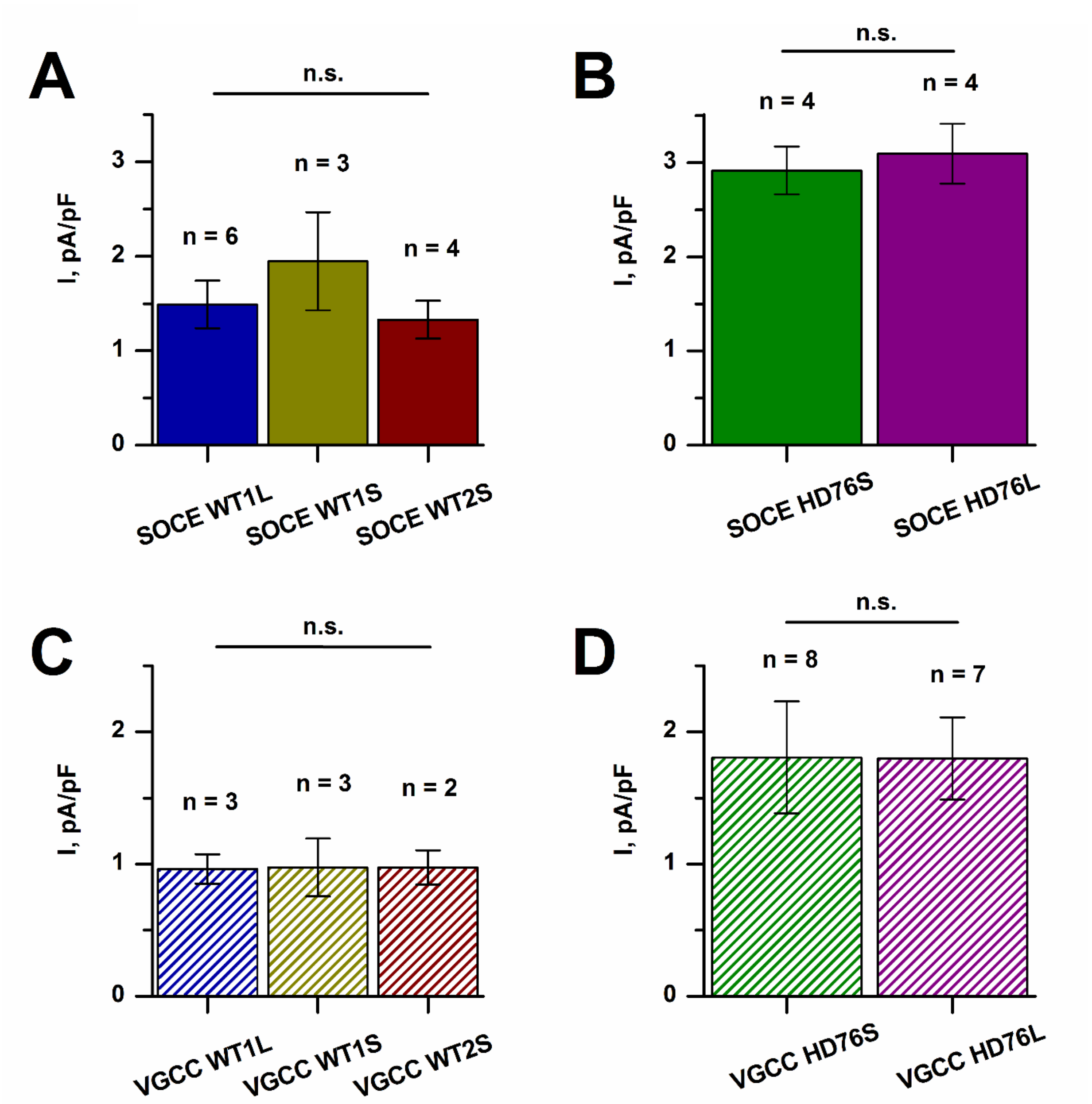

Figure S1. Electrophysiological characteristics of different WT and HD76 cell lines. Average amplitudes of the full developed normalized SOC currents (A and B, solid filling) determined at a test potential of –80 mV and VGCC maximal currents (C and D, dense filling) for (**A, C**) WT1L (blue), WT1S (dark yellow) and WT2L (wine) GABA MSNs and (**B, D**) HD76 obtained by using lentiviral transduction method (HD76L, purple) or Sendai viruses (HD76S, green). The amplitudes are normalized on the cells capacitance and plotted as the mean ± SEM (n = number of single cell experiments). n.s. indicates the absence of statistically significant differences. The cell lines are represented in Table S1.

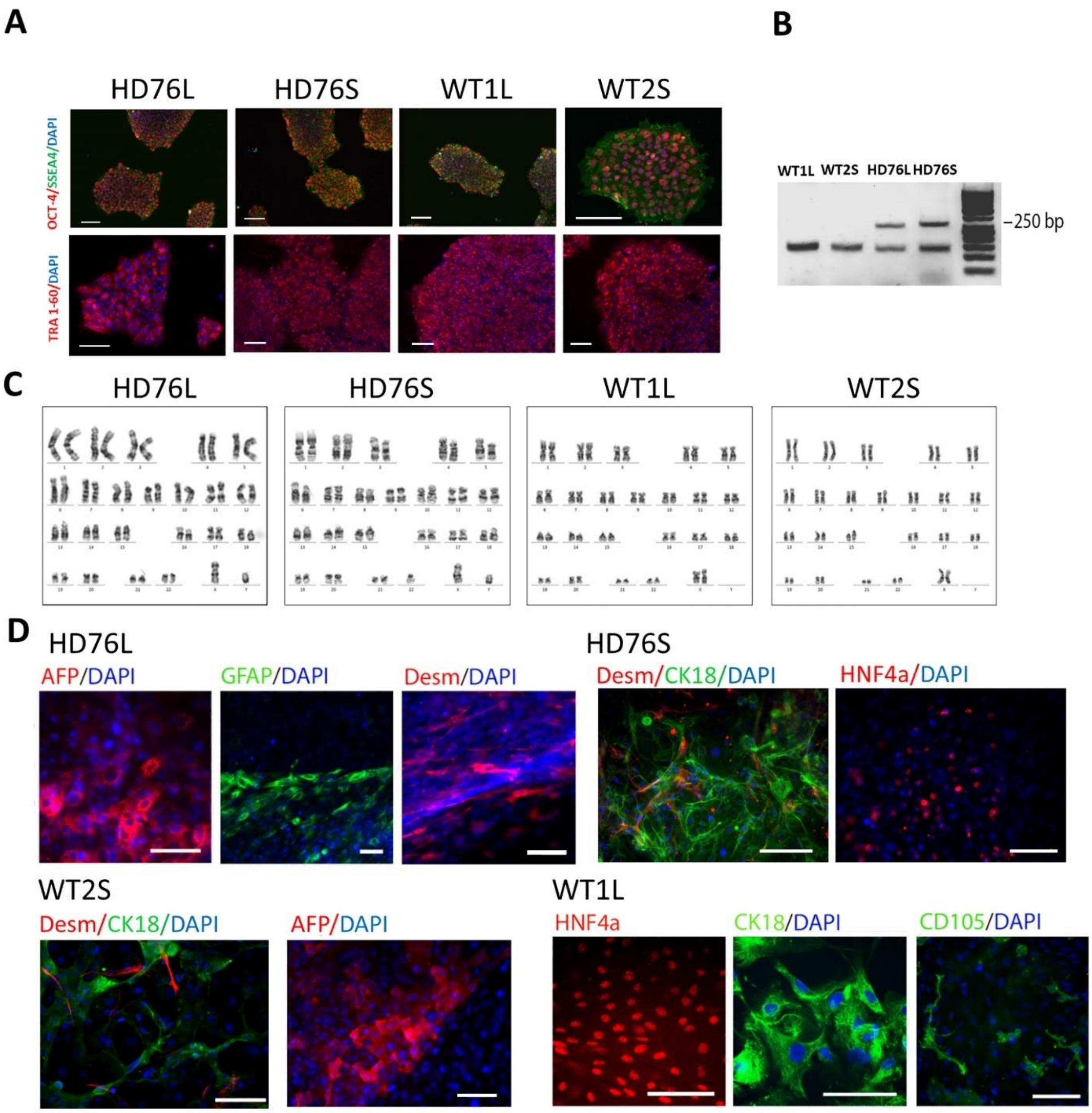

Figure S2. PSC lines characterization. HD76L (juvenile HD-specific) and WT1L (wild type) – neuronal lines differentiated from iPSCs obtained by lentiviral approach; HD76S and WT2S – neuronal lines differentiated from iPSCs obtained by Sendai virus approach (**A**) Immunohistochemical analysis for OCT4, Nanog, SSEA-4 and TRA1-60 expression in iPSC lines, nuclei are counterstained with DAPI (blue), scale bar 100 μm. (**B**) PCR analysis of the CAG-repeat length in iPSC lines. (**C**) Karyotype of iPSC lines by GTG-banding. (**D**) Representative images of spontaneously differentiated iPSHD and iPS cell lines immunostained for ectoderm (cytokeratin 18, GFAP), mesoderm (CD105, desmin, CD31), and endoderm (α-fetoprotein, HNF4a) markers, nuclei are counterstained with DAPI (blue), scale bar 100 μm.

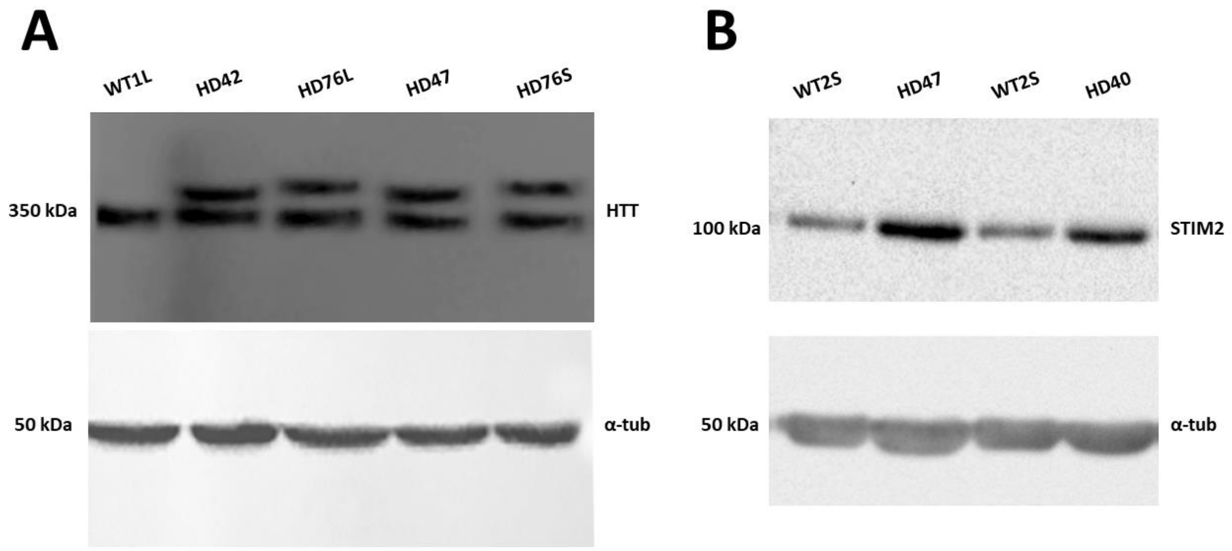

Figure S3. (A) HTT is overexpressed in mutant neurons. Representative Western blot showing the expression level of HTT in neurons derived from WT1L, HD42, HD76L, HD47, HD76S. Lower panel represents loading control (α-tubulin). **(B)** STIM2 is overexpressed in low-repeat HD models. Representative Western blot showing the expression level of STIM2 in neurons derived from WT2S, HD40 and HD47. The cell lines are represented in Table S1.

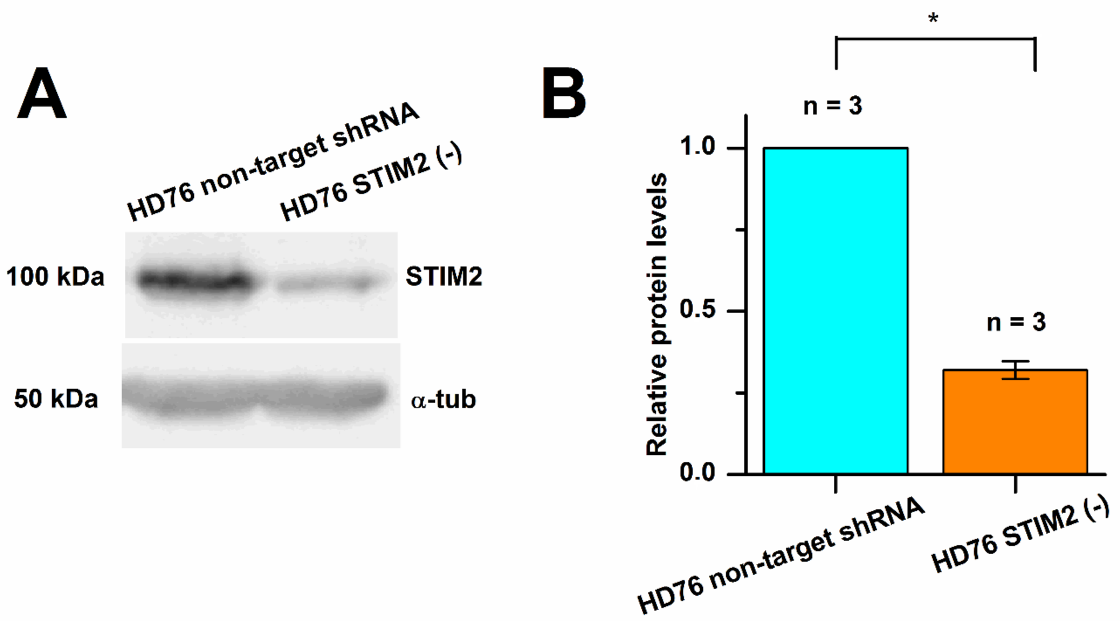

Figure S4. Lentiviral transfection efficiency. (**A**) Representative Western blot showing the expression level of STIM2 in HD76 GABA MSNs expressing non-target shRNA (HD76 non-target shRNA) or shRNA against STIM2 (HD76 STIM2(-)). (**B**) Relative STIM2 levels in HD76 GABA MSNs expressing non-target shRNA (HD76 non-target shRNA, cyan bar) or shRNA against STIM2 (HD76 STIM2(-), orange bar). Biological replicates are mentioned above the bars. The amplitudes are plotted as the mean ± SEM. The asterisk indicates that differences in amplitudes are statistically significant ($p < 0.05$)

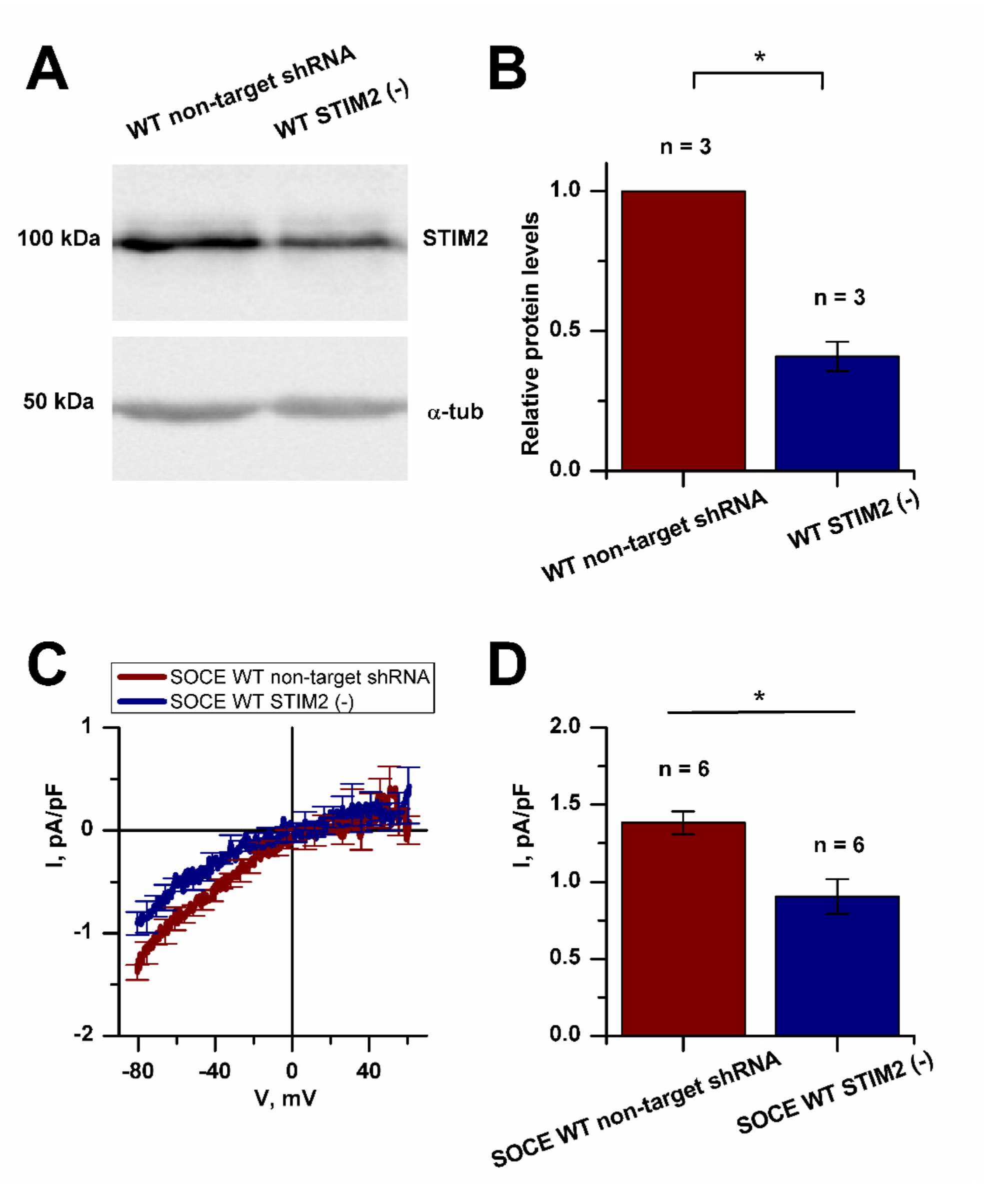

Figure S5. STIM2 mediates SOCE in WT GABA MSNs. (**A**) Representative Western blot showing the expression level of STIM2 in WT GABA MSNs expressing non-target shRNA WT non-target shRNA) or shRNA against STIM2 (WT STIM2(-)). (**B**) Relative STIM2 levels in WT GABA MSNs expressing non-target shRNA WT non-target shRNA, wine bar) or shRNA against STIM2 (HD76 STIM2(-), blue bar). Biological replicates are mentioned above the bars. The amplitudes are plotted as the mean ± SEM. The asterisk indicates that differences in amplitudes are statistically significant ($p < 0.05$). (**C**) Average Current-Voltage relationships (I-V curves) of normalized currents evoked by passive depletion of calcium stores with thapsigargin (1µM) in WT GABA MSNs expressing non-target shRNA (WT non-target shRNA, wine line), WT expressing shRNA against STIM2 (WT STIM2(-), blue line), The number of experiments is depicted at the panel (D). (D) Average amplitude or the normalized SOC currents determined at a test potential of –80 mV for WT GABA MSNs expressing non-target shRNA (wine) or expressing shRNA against STIM2 (blue). The amplitudes are plotted as the mean ± SEM (n = number of single cell experiments). The asterisk indicates that differences in amplitudes are statistically significant ($p < 0.05$).

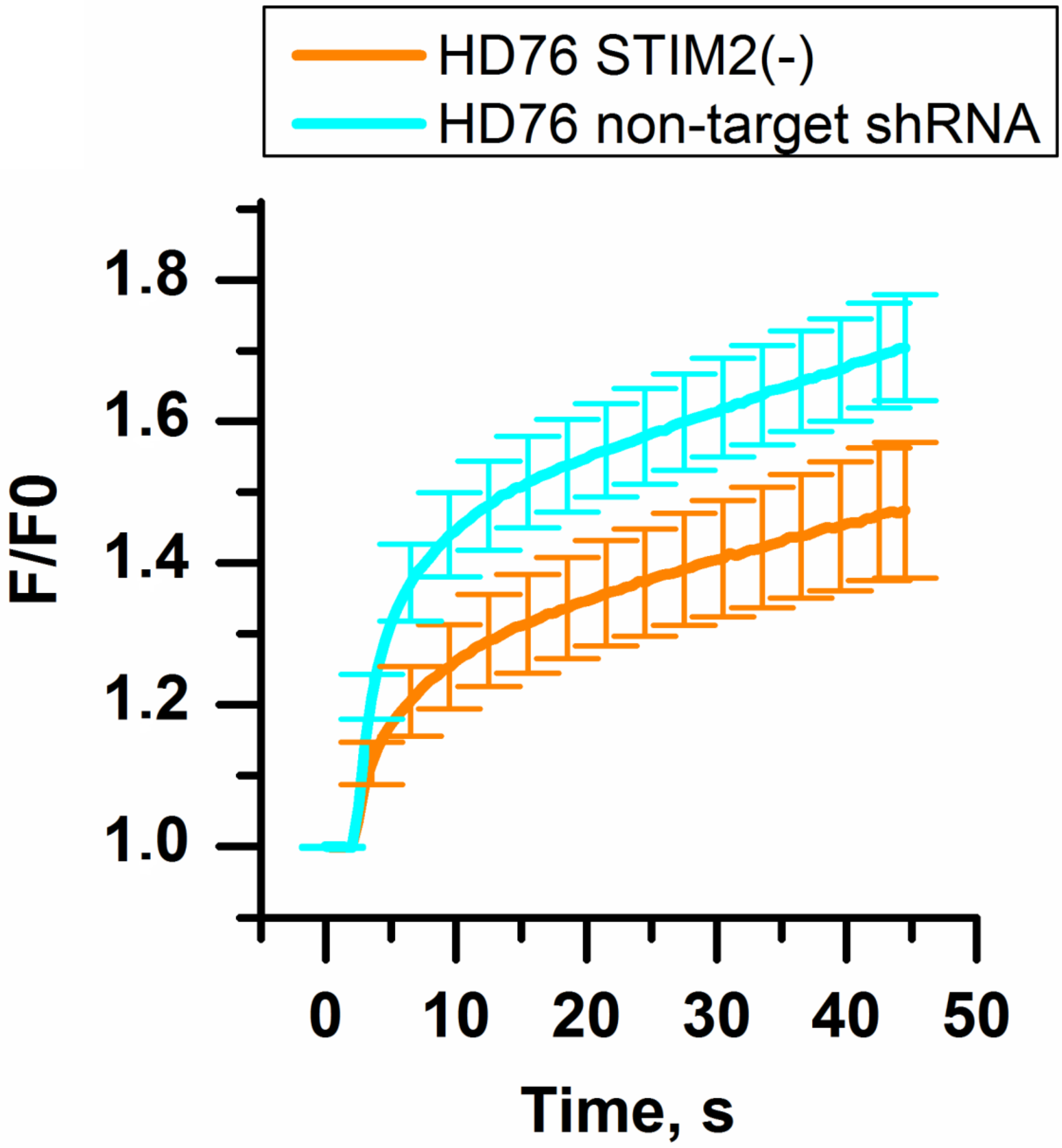

Figure S6. Fluorescent calcium imaging in HD76 STIM2(-). Relative fluorescence of Fluo-4 calcium dye after returning the 4 mM Ca2+ into the bath solution and application of 1μM thapsigargin to prevent refilling the stores in HD76 expressing non-target shRNA (HD76 non-target shRNA, cyan line, n=16), and HD76 expressing shRNA against STIM2 (HD76 STIM2(-), orange line, n=6). The amplitudes are plotted as the mean ± SEM.

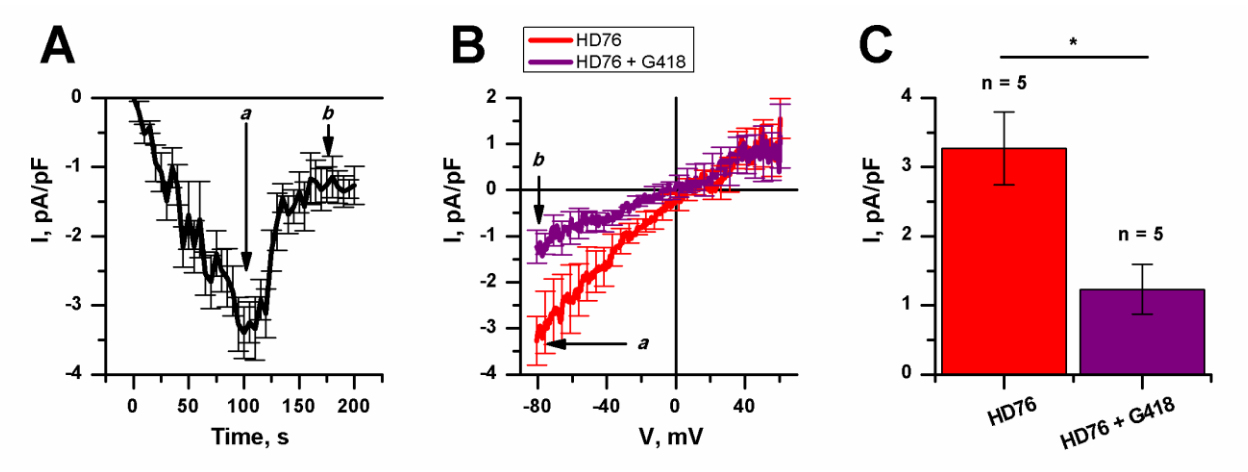

Figure S7. Effect of G418 on SOCE in HD76 neurons. **(A)** Amplitudes of thapsigargin-induced store-operated calcium currents in HD76 neurons at a test potential of –80 mV plotted as a function of time. **(B)** Average I-V curves of normalized thapsigargin-induced calcium currents in HD76 neurons before (red line, marked *a*) and after application of 50 μM G418 (violet line, marked *b*). **(C)** Average amplitudes of thapsigargin-induced calcium currents at the potential of -80 mV in HD76 neurons before (red) and after application of 50 μM G418 (violet). The amplitudes are plotted as the mean ± SEM (n = number of single cell experiments). The asterisks indicate that differences in amplitudes are statistically significant ($p < 0.05$).

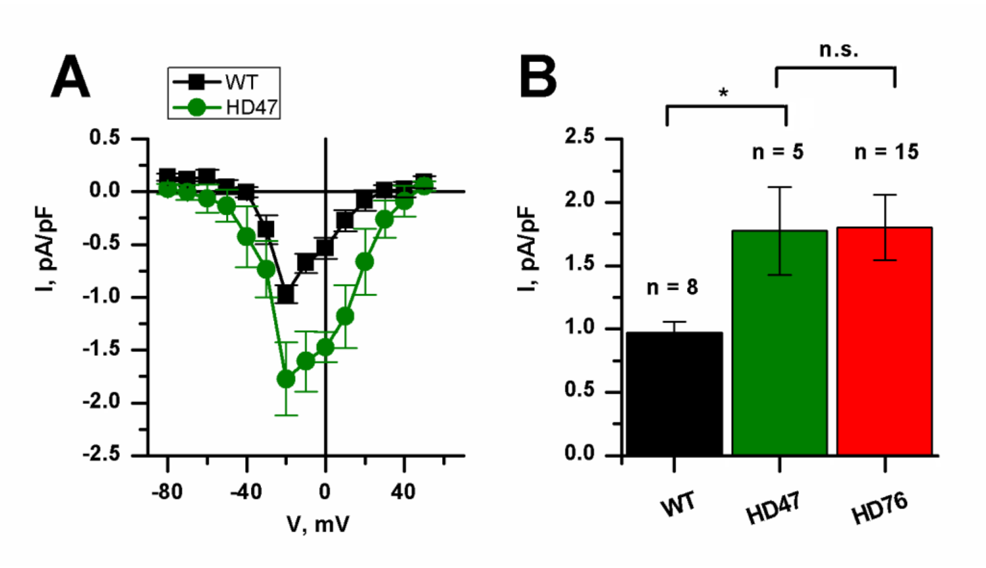

Fig. S8 Disturbance of VGCC in HD47 neurons. **(A)** Average I-V curves of normalized voltage-gated calcium currents for HD47 (green circles) and WT (black squares) GABA MSNs. The number of experiments is depicted at the panel (B) **(B)** Average amplitude of VGCC currents at the potential of -20 mV for HD76 (red), HD47 (green) and WT (black) GABA MSNs. The amplitudes are plotted as the mean ± SEM (n = number of single cell experiments). The asterisk indicates that differences in amplitudes are statistically significant ($p < 0.05$); n.s. indicates the absence of statistically significant differences ($p > 0.05$).

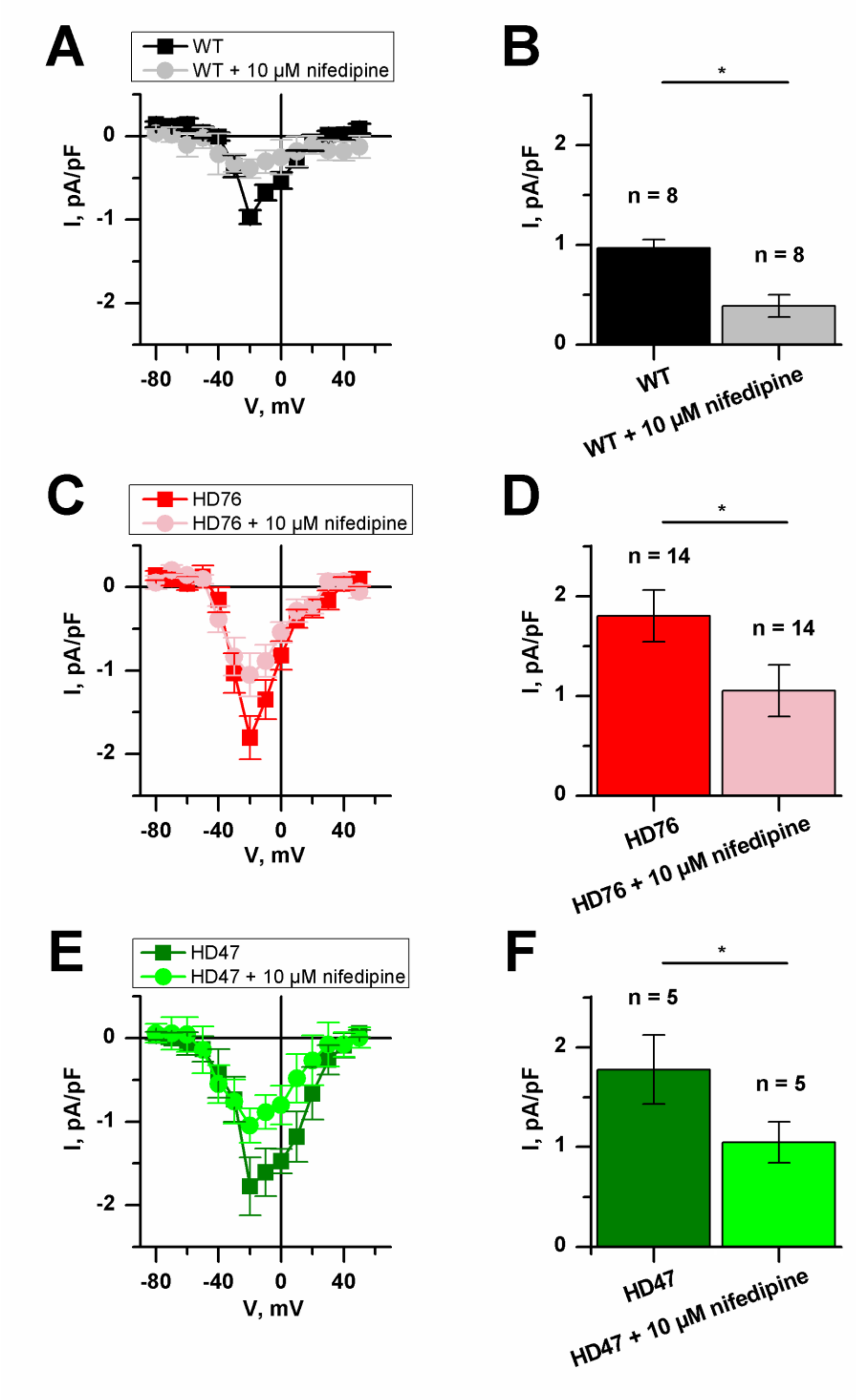

Figure S9. Nifedipine sensitivity of voltage-gated calcium channels. Average I-V curves of normalized voltage-gated calcium currents in (**A**) WT GABA MSNs before (black squares) and after application of 10 µM nifedipine (gray circles); (**C**) HD76 before (red squares) and after application of 10 µM nifedipine (magenta circles); (**E**) HD47 before (olive squares) and after application of 10 µM nifedipine (green cicles); The numbers of experiments for (A), (C) and (E) are depicted at the panels (B), (D) and (F) respectively (**B, D, F**) Average amplitudes of VGCC currents at the potential of -20 mV for WT (B) HD76 (D) and HD47 (F) GABA MSNs before (left bars) and after application of 10 µM nifedipine (right bars). The amplitudes are plotted as the mean ± SEM (n = number of single cell experiments). The asterisks indicate that differences in amplitudes are statistically significant ($p < 0.05$).

Table S1. iPS cell lines used in the study

Line name	Type of cell line	Origin info	Method of reprogramming	Morphology	Phenotype	Genotype	Mutation analysis (IF APPLICABLE)	Microbiology and virology	Differentiation potential
**Analysis**				*Microscopy*	*RT-PCR and immunocytochemistry*	*Karyotype (G-banding)*	*PCR analysis for CAG-expansion*	*Mycoplasma testing by PCR*	*Embryoid body formation*
**HD76S (HD76)**	iPSC	**HD patient**<br>Age: 17 years<br>Sex: male<br>Ethnicity: Caucasian<br>**Repeats in expanded allele: 76**	Sendai virus	Typical morphology for human pluripotent stem cells	ICC: Oct4, Nanog, SSEA4, TRA-1-60	46XY<br>Resolution: 400 bands	Heterozygote with one expanded allele	negative	ICC: Desmin, CK18, HNF4α
**HD76L (HD76)**	iPSC		Lentivirus	Typical morphology for human pluripotent stem cells	ICC: Oct4, SSEA4, TRA-1-60	46XY<br>Resolution: 400 bands	Heterozygote with one expanded allele	negative	ICC: AFP, CD105, Desmin
**iPSHD22 (HD47)**	iPSC	**HD patient**<br>Age: N/A<br>Sex: female<br>Ethnicity: Caucasian<br>**Repeats in expanded allele: 47**	Lentivirus	(Nekrasov et al., 2016)					

**iPSHD34**<br><br>**(HD42)**	iPSC	**HD patient**<br><br>Age: N/A<br><br>Sex: female<br><br>Ethnicity: Caucasian<br><br>**Repeats in expanded allele: 42**	Lentivirus	(Nekrasov et al., 2016)						
**iPSHD11**<br><br>**(HD40)**	iPSC	**HD patient**<br><br>Age: N/A<br><br>Sex: female<br><br>Ethnicity: Caucasian<br><br>**Repeats in expanded allele: 40**	Lentivirus	(Nekrasov et al., 2016)						
**WT1L**	iPSC	**Healthy donor**<br><br>Age: 26 years<br><br>Sex: female<br><br>Ethnicity: Caucasian	Lentivirus	Typical morphology for human pluripotent stem cells	ICC: Oct4, Nanog, Tra-1-60, SSEA-4	46XX<br><br>Resolution: 400 bands	N/A	negative	ICC: HNF4α, CK18, CD105, EPCAM,	

**WT1S**	iPSC	**Healthy donor**<br><br>Age: 26 years<br><br>Sex: Female<br><br>Ethnicity: Caucasian	Sendai virus	Typical morphology for human pluripotent stem cells	ICC: Nanog, SSEA-4, TRA-1-60	46XX<br><br>Resolution: 400 bands	N/A	negative	ICC: CD105, CD31, CK18, AFP
**WT2S**<br><br>**(UEF3B)**	iPSC	**Healthy donor**<br><br>Age: 60 years<br><br>Sex: male<br><br>Ethnicity: Caucasian	Sendai virus	(Holmqvist et al., 2016)					

Table S2. Primer sequences

TARGET	APPLICATION	FORWARD/REVERSE PRIMERS 5'-3'
HUNTINGTIN	CAG-expansion validation	F: CCTTCGAGTCCCTCAAGTCCTTC<br>R: GGCTGAGGAAGCTGAGGAG
OCT4	Pluripotency marker	F: CGACCATCTGCCGCTTTGAG<br>R: CCCCCTGTCCCCCATTCCTA
SOX2	Pluripotency marker	F: AACCAGCGCATGGACAGTTA<br>R: GACTTGACCACCGAACCCAT
SALL4	Pluripotency marker	F: TGGCGGAGAGGGCAAATAAC<br>R: ATGCTGAAGAACTCCGCACA
DPPA5	Pluripotency marker	F: AAGATGGGAACTCTCCCGGC<br>R: GCAAGTTTGAGCATCCCTCGC
HESX1	Pluripotency marker	F: CCCCCTGGTGTAGCCATTAGT<br>R: ACCATCTTTCCCTGATGAGCTG
NANOG	Pluripotency marker	F: CAGCCCTGATTCTTCCACCAGTCCC<br>R: TGGAAGGTTCCCAGTCGGGTTCACC
FOXD3	Pluripotency marker	F: CTCGTACATCGCGCTCATCA<br>R: TGTAAGCGCCGAAGCTCT
GAPDH	Housekeeping/reference	F: GAAGGTGAAGGTCGGAGTCA<br>R: GCAAGTTTGAGCATCCCTCGC

Table S3. Antibodies

ANTIGEN,<br>FUNCTION	HOST SPECIES	DILUTION	COMPANY CAT # and RRID
NANOG,<br>Pluripotency marker	Rabbit	1:800	Abcam Cat# ab21624
SSEA 4,<br>Pluripotency marker	Mouse	1:40	DSHB Cat# MC-813-70
TRA-1-60,<br>Pluripotency marker	Mouse	1:150	Abcam Cat# ab16288
OCT4,<br>Pluripotency marker	Rabbit	1:400	Abcam Cat# ab18976
CD105,<br>Mesoderm marker	Mouse	1:100	Agilent Cat# M3527
CK7,<br>Ectoderm marker	Mouse	1:100	Agilent Cat# M7018,
CD31,<br>Mesoderm marker	mouse	1:200	BD Pharmingen<br>Cat# 555444
GFAP,<br>Ectoderm marker	Mouse	1:100	Abcam Cat# ab10062
AFP,<br>Endoderm marker	Mouse	1:100	Abcam Cat# ab54745
Desmin,	Rabbit	1:600	Abcam Cat# ab15200

Mesoderm marker			
HNF4α,<br><br>Endoderm marker	Rabbit	1:100	Abcam Cat# ab92378
CK18,<br>Ectoderm marker	Mouse	1:150	Agilent Cat# M7010
DARPP-32,<br><br>Marker of GABA-ergic neurons	Rabbit	1:80	Abcam Cat# ab40801
STIM2	Rabbit	1:1000	Cell Signaling Technology Cat# 4917
HTT	Rabbit	1:5000	Abcam Cat# ab109115,
α-tubulin	Mouse	1:1000	Sigma Cat# T6074
